# N-glycosylation patterns of plasma immunoglobulin G in anti-synthetase syndrome disease

**DOI:** 10.3389/fimmu.2025.1538219

**Published:** 2025-06-18

**Authors:** Jing Zhao, Yanhong Li, Yingying Ling, Tong Wu, Yinlan Wu, Chunyu Tan, Lu Cheng, Deying Huang, Yi Liu, Yong Zhang

**Affiliations:** Department of Rheumatology and Immunology, Laboratory of Rheumatology and Immunology, Institutes for Systems Genetics, West China Hospital, Sichuan University, Chengdu, China

**Keywords:** autoimmune disease, anti-synthetase syndrome (ASS), N-glycosylation, intact N-glycopeptide, immunoglobulin g (IgG)

## Abstract

**Introduction:**

Anti-synthetase syndrome (ASS) is a subtype of idiopathic inflammatory myopathy (IIM) characterized by characteristic rash, myositis, and interstitial lung disease (ILD). The etiology of ASS is unknown, and patients have a poor quality of life and are prone to pulmonary infection. Recent studies have elucidated the potential role of abnormal glycosylation of immunoglobulin G (IgG) in the pathogenesis of autoimmune diseases. However, the pattern of patient-specific IgG N-glycosylation in ASS has not been fully elucidated.

**Methods:**

the GlycoQuant method was used to quantify the intact N-glycopeptides of IgG from 30 ASS patients and 30 healthy controls (HCs).

**Results and Discussion:**

Thirteen differentially expressed intact N-glycopeptides were identified (p<0.05). Notably, we observed increased fucosylation (p<0.0001) and decreased N-acetylneuraminic acid (p<0.05) in ASS patients. In addition, specific glycosylation patterns correlated with lung function parameters. Our study revealed the IgG glycosylation profile in ASS patients and provided a valuable reference for further investigation of its potential diagnostic and prognostic applications.

## Introduction

Anti-synthetase syndrome (ASS) represents a distinct clinical type of idiopathic inflammatory myopathy (IIM), characterized by myopathic manifestations (muscle weakness and myalgia) and systemic involvement, including characteristic rash, arthritis, interstitial lung disease (ILD), and cardiac complications. A defining feature of ASS is the presence of anti-transfer RNA synthetases (ARSs) (1). The enzyme family includes anti-Jo-1, anti-PL-7, anti-PL-12, anti-EJ, anti-OJ, anti-KS, anti-Zo, anti-Ha, anti-JS, anti-SC, and anti-YRS, but data on the screening of anti-ARS in non- systemic autoimmune rheumatic disease (SARD) patients are often limited (2). The detection of certain anti-ARS agents, such as antibodies against PL-12 and KS in patients with idiopathic ILD has been reported (3, 4). One study reported that 10% of patients with idiopathic ILD had anti-ARS (5). At present, the pathogenesis of ASS is not clear, the quality of life of ASS patients has decreased, and the prognosis of patients with ILD has worsened. Therefore, in addition to the anti-ARS family, other in-depth studies are urgently needed to enhance the understanding of ASS and find new therapeutic targets (6).

Immunoglobulins (Igs) are a class of glycoproteins that play critical roles in immune function (7, 8). Their Fc region, which is responsible for their effector functions, is significantly influenced by the attached N-glycans. Among the five isotypes (IgM, IgD, IgG, IgA, and IgE) of Igs, IgG is the most prevalent serum autoantibody found in autoimmune disorders (9). Research has shown that variations in the glycosylation patterns of IgG are linked to various diseases (10, 11). A decrease in galactosylation is correlated with heightened inflammatory responses, and this connection is evident in the progression or flare-ups of autoimmune diseases (12, 13), such as systemic lupus erythematosus (SLE) (14), rheumatoid arthritis (RA) (15), inflammatory bowel disease (IBD) (16, 17), Sjögren’s syndrome (SS) (18), anti-neutrophil cytoplasmic antibody (ANCA)-associated vasculitis (AAV) (19), myasthenia gravis and Guillain–Barré syndrome (20). Similar patterns have been observed in chronic infectious diseases such as tuberculosis (12), HIV infection (21), and hepatitis B and C virus infection (22). Therefore, analyzing IgG glycosylation offers a promising avenue for predicting the severity of these diseases (23, 24). Earlier investigations revealed the distinct FC-glycan profiles of IgGs in ASS patients with anti-Jo1 antibodies compared with those in healthy individuals (25). However, a detailed quantitative exploration of the site-specific N-glycosylation of IgGs in the peripheral blood of ASS patients remains unexplored. This study aimed to explore a novel aspect of ASS by examining global IgG glycosylation patterns and provide a new perspective on immune dysregulation in ASS, which current antibody-based studies may not fully capture.

The specificity and complexity of glycosylation patterns make them promising biomarkers, but accurate measurement of intact N-glycopeptides in many clinical samples is highly challenging. A quantitative pipeline, GlycoQuant, has been confirmed to effectively identify intact N-glycopeptides, which combines mass spectrometry fragmentation technology (EThcD-sceHCD) with intact N-glycopeptide batch quantification software (PANDA v.1.2.5). In this study, we attempted to identify the N-glycosylation pattern of plasma IgG in ASS patients via this method and lay a framework for future mechanistic investigations.

## Materials and methods

### Biospecimen collection

Our study identified and recruited thirty patients diagnosed with ASS within the years 2021 and 2022 from the Department of Rheumatology, West China Hospital, Sichuan University. Patients with malignancies, serious infections, or any other autoimmune disease were excluded from this study. The diagnosis of ASS for each participant was rigorously determined following the diagnostic guidelines established by Solomon et al. in 2011 (26), and a diagnosis of ASS was confirmed if a patient tested positive for anti-ARS antibodies and met either two major criteria or one major criterion alongside two minor criteria. The major criteria include ILD (with exclusions for other causes) and polymyositis/dermatomyositis. The minor criteria include arthritis, Raynaud’s phenomenon, and mechanic’s hands. The diagnosis of myositis must adhere to the criteria recommended by Bohan and Peter in 1975, which are: symmetrical proximal muscle weakness, elevated serum muscle enzymes, electromyography indicating muscle origin damage, and a muscle biopsy supporting the diagnosis of inflammatory myopathy. Confirmation of the diagnosis is achievable by meeting any three of these four criteria. This research was granted approval by the Ethics Committee of West China Hospital, Sichuan University, and all participants provided informed consent prior to specimen collection. We selected 30 healthy controls (HCs) from the family members of the hospitalized patients whose age and sex did not differ from those of the ASS patients. A 5-milliliter sample of peripheral blood was obtained and stored in tubes containing ethylenediaminetetraacetic acid disodium salt (EDTA). Following collection, these samples were centrifuged at 1,200 g for 10 minutes at 4 °C. This process was aimed at isolating the plasma, which was then preserved at -80 °C, until further analysis.

### Clinical and laboratory data collection

The necessary data were retrospectively obtained from patient medical records. These data included demographic characteristics, medical history, complications, comorbidities, medication utilization, blood routine parameters, liver and kidney function assessments, autoantibody profiles, lung function measurements, and inflammatory markers.

### Isolation, purification and digestion

The process of isolating, purifying, and digesting IgGs is a detailed procedure that involves several precise steps (27). Briefly, 40 μL of immobilized protein A/G agarose and 200 μL of binding buffer were incubated with 20 μL of plasma on a rotator for 2 h at 4°C. Next, any proteins that were not bound were washed away with 500 μL of binding buffer. Subsequently, 50 μL of elution buffer containing 0.1 M formic acid was added to release the IgGs. To neutralize the mixture, 50 μL of neutralization buffer was added until the mixture reached a physiological pH. The protein concentration was subsequently quantified via a bicinchoninic acid (BCA) protein assay (Thermo Fisher Scientific, USA). Purified IgGs (20 μg) were denatured by heating at 95°C for 10 min, followed by reduction with 20 mM dithiothreitol (DTT) at 56°C for 30 min and alkylation with 50 mM iodoacetamide (IAA) at 25°C in the dark for 30 min. The mixture was transferred to a 30 kDa ultrafiltration tube (Millipore, Bedford, MA, USA) and replaced with 50 mM ammonium bicarbonate (NH_4_HCO_3_) buffer (pH 8.5). Finally, 0.5 μg of sequence-grade trypsin (Promega, Madison, WI, USA) was added, and the mixture was incubated for 2 h at 37°C. The obtained IgG peptides were quantified via peptide colorimetry (Thermo Fisher Scientific, USA).

### LC-MS/MS analysis

As previously described (27), we utilized an advanced EASY-nLC 1200 HPLC system connected to an Orbitrap Fusion Lumos mass spectrometer (Thermo Fisher Scientific, USA) to conduct peptide analysis. Specifically, the peptides were dissolved in buffer A (0.1% FA in water) at a flow rate of 350 nL/min and separated on a 20-centimeter column (ReproSil-Pur C18-AQ, 1.9 μm, 75 μm inner diameter; Dr Maisch) with a 30-min gradient. The separated samples were analyzed via EThcD-sceHCD-MS/MS. The parameter settings were outlined following previously established methods (28).

### Data analysis and bioinformatics

Intact N-glycopeptides were identified via Byonic software (version 3.10, Protein Metrics, Inc.) targeting the human IgG database with a mass tolerance of ±6 parts per million (ppm) for precursor ions and ±20 ppm for fragment ions. Up to two cleavage sites were allowed for trypsin digestion. Fixed modifications included amino methylation (C), whereas variable modifications consisted of oxidation (M) and acetylation (N-term). Additionally, 182 human N-glycans were designated N-glycan modifications. The proteome was filtered to a false discovery rate of 1%, and confident intact N-glycopeptides required a score of at least 200 and at least six amino acids. All glycopeptide profile matches (GPSMs) were performed manually. The quantification of intact N-glycopeptides was conducted via PANDA software (v 1.2.5) in label-free quantification mode with default values used for all other parameters. The selection of highly reliable and reproducible N-glycans is based on three main criteria: 1) analytical reproducibility across multiple sample preparations and instrument runs, where glycans with coefficient of variation (CV) values less than 15% in repeated measurements are prioritized; 2) statistical significance in their abundance differences between ASS patients and controls, with a *p*-value threshold of less than 0.05 after adjusting for multiple comparisons via the false discovery rate (FDR) method; and 3) biological relevance, where we refer to literature on the role of specific N-glycans in immune-related processes and autoimmune diseases.

R programming software was utilized for data analysis. The differentially expressed intact N-glycopeptides were identified via a moderated t test in the limma package following median normalization of the data. Additionally, R programming software and GraphPad Prism 9.5 software were used for data visualization. The supplementary data were analyzed via SigmaPlot 12.5, GraphPad Prism 9.5, and SPSS Statistics 29.0.1.09(171). The results are presented as the mean ± standard deviation (M ± SD) or median (Q25, Q75), with count data expressed as n (%). Intergroup comparisons were conducted via Student’s t test. Correlation analyses employ Pearson or Spearman coefficients with the choice of test determined by data distribution, followed by multivariate analysis via the appropriate regression model (linear or binary logistic). A *p*-value of less than 0.05 was considered statistically significant. Statistical differences between the ASS and HC groups are indicated as follows: *p<0.05, **p<0.005, ***p<0.001, **** *p*<0.0001.

## Results and discussion

### Experimental design

**Figure 1:**
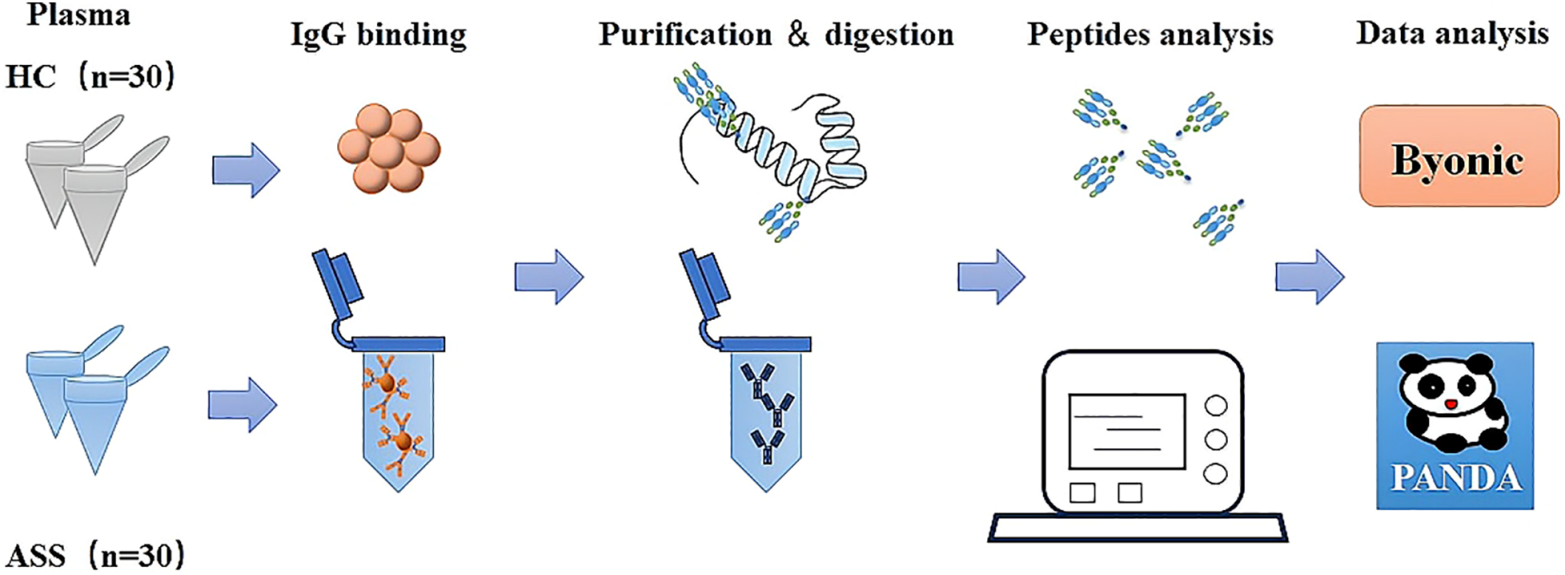
Schematic illustration of the workflow for the analysis of IgG intact N-glycopeptides via the GlycoQuant method.

This study profiles the IgG glycosylation patterns in patients with anti-synthetase syndrome (ASS) and explores their clinical and laboratory correlations through a systematic workflow (**Figure 1**). To do this, plasma IgG molecules were isolated, purified, and digested from 30 ASS patients and 30 healthy controls (HCs). All the peptides were subsequently analyzed via the mass spectrometry technique EThcD-sceHCD-MS/MS. Finally, the intact N-glycopeptides were identified with Byonic software and quantified via PANDA software. This study shed light on the patterns of IgG glycosylation in ASS and its potential implications for understanding this disease.

### Baseline demographics and clinical characteristics

The demographic and clinical characteristics of the study participants are presented in detail in **Supplementary Table S1**. The ASS group consisted of 30 patients, and the HC group also included 30 participants. The average age of the HC group was 50 ± 4 years, while that of the ASS group was 50 ± 8 years. The median disease duration among ASS patients was 12 months, and their average body mass index (BMI) was 23.238 ± 3.657 kg/m (2). Among the participants, 4 had hypertension, 3 had diabetes, and 7 had hyperlipidemia. The mean myositis disease activity assessment visual analogue scale (MYOACT) score was 0.197 ± 0.094, and the mean myositis intention to treat activity index (MITAX) score was 0.181 ± 0.149. The clinical manifestations included rash in 26.7% of the cases, Mechanic’s hand in 40%, muscle weakness in 23.3%, myalgia in 40%, dyspnea in 73.3%, cough in 86.7%, expectoration in 73.3%, arthralgia in 43.3%, the Velco sign in 30%, and Raynaud’s phenomenon in 10%. In terms of the antibody test results, 16 patients were positive for anti-jo-1 antibody; 7 patients were positive for anti-PL7 antibody; 5 patients were positive for anti-PL12 antibody; 3 patients were positive for anti-EJ antibody; 1 patient was positive for anti-OJ antibody; and 1 patient was positive for anti-HA antibody. All participants were administered glucocorticoids, with a mean dose of 43.5 mg, and 22 of them also received cyclophosphamide.

### N-glycosylation patterns of plasma IgG in patients with ASS

**Figure 2:**
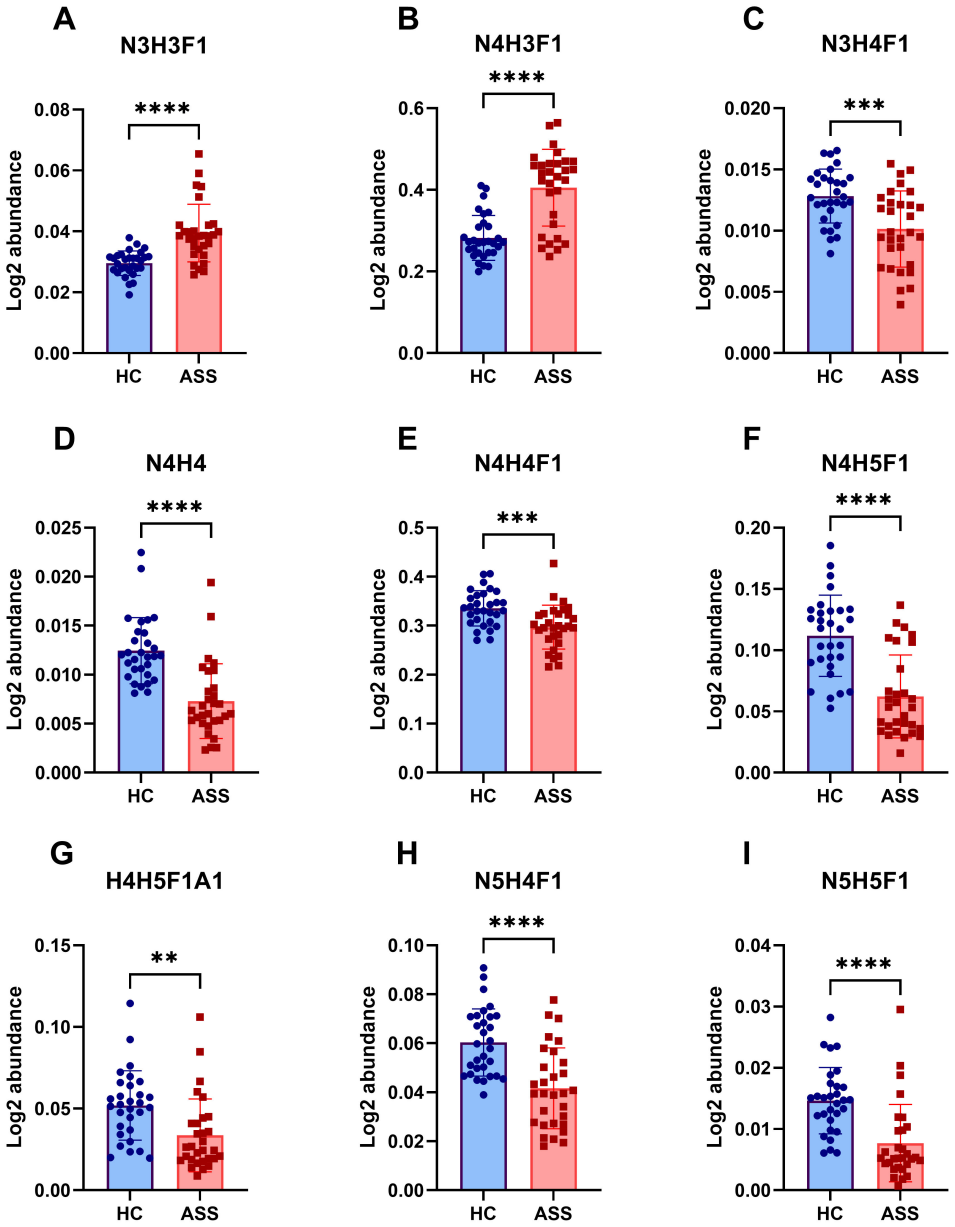
Differences in IgG N-glycans between the ASS and HC groups. This figure highlights the significant variations in N-glycosylation patterns observed between the ASS and HC groups. **(A)** N3H3F1, **(B)**N4H3F1, **(C)**N3H4F1, **(D)** N4H4, **(E)** N4H4F1, **(F)** N4H5F1, **(G)** N4H5F1A1, **(H)** N5H4F1, **(I)** N5H5F1. H, hexose; N, N-acetylhexosamine; F, fucose; A, N-acetylneuraminic acid. **p<0.005, ***p<0.001, ****p<0.0001.

**Figure 3:**
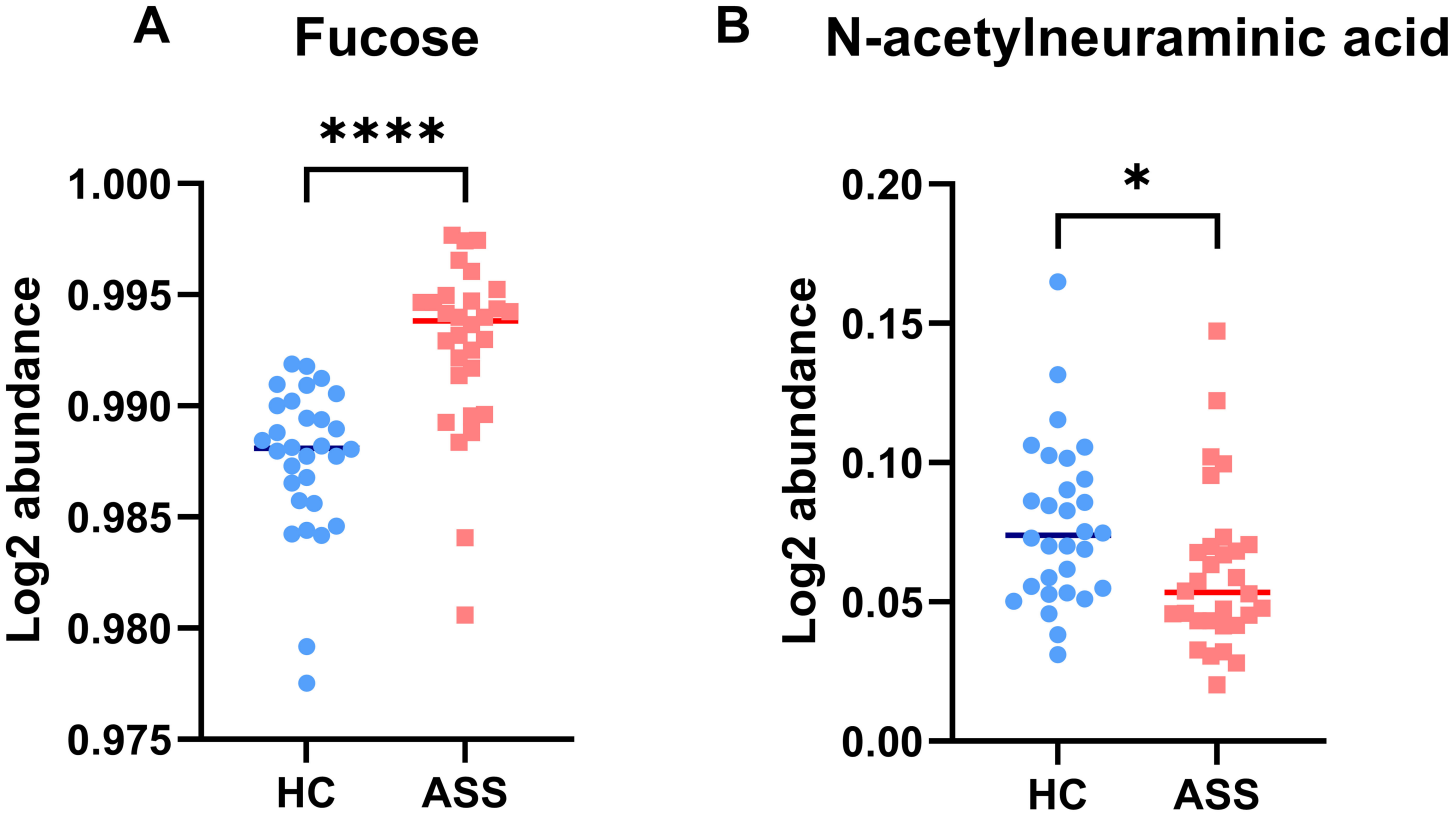
Characteristics of IgG N-glycans containing fucose **(A)** and N-acetylneuraminic acid **(B)** in the ASS and HC groups. *p <0.05, **** *p*<0.0001.

We conducted qualitative and quantitative analyses of the IgG intact N-glycopeptides from the ASS and HC groups. A total of 11 highly reliable and reproducible N-glycans were quantified in both groups (**Supplementary Table S2**). The quantitative results of the IgG N-glycans in these two groups were subsequently compared. As shown in **Figure 2**, in comparison with those in HCs, 2 N-glycans were increased (**Figures 2A, B**) and 7 were decreased (**Figures 2C-I**) among patients with ASS. In addition, the levels of the 2 N-glycans did not differ significantly between the two groups (**Supplementary Figure S1**). In addition, we selected N-glycans containing F (fucose) and A (N-acetylneuraminic acid) from the 11 highly reliable and reproducible N-glycans, added them and found that fucose was significantly increased, whereas N-acetylneuraminic acid was significantly decreased in ASS patients (**Figure 3**). The binding of IgG to FcγRIIIA on NK cells activates antibody-dependent cell cytotoxicity (ADCC) (23). In *in-vitro* models using monoclonal antibodies and affinity studies employing surface plasmon resonance, afucosylated antibodies have been demonstrated to possess an affinity for FcγRIIIA that is up to 100-fold greater than that of fucosylated antibodies, regardless of the subclass (29, 30). In the field of therapeutics, afucosylation serves as an essential means of enhancing the capacity of monoclonal antibodies to induce ADCC and thereby improving treatment efficacy (31). In contrast to previous findings that afucosylation promotes inflammation in *in-vitro* models, some studies have reported an increase in fucosylation among JO-1^+^ myositis patients (25), SLE patients (32) and RA patients (33), which is consistent with our study. Notably, the level of fucosylation in SLE patients was positively correlated with the SLEDAI score, suggesting that the influence of disease status and/or disease type on the effect of N-glycosylation requires further investigation. Our study also revealed a reduction in sialylation among ASS patients, which is in line with findings from other studies on autoimmune diseases (14, 15). Sialylation is widely acknowledged for its anti-inflammatory properties, and its impact on the effector function of IgG has been extensively investigated. Sialylated IgG interacts with CD23, a C-type lectin receptor, leading to the upregulation of inhibitory FcγRIIB on B cells and triggering a negative signaling cascade that counteracts the activating signal following BCR ligation. Moreover, sialylated IgG also engages with CD22, inducing a tolerogenic state in dendritic cells and resulting in the generation of regulatory T (Treg) cells expressing forkhead box P3^+^ that suppress immune responses (23).

**Figure 4:**
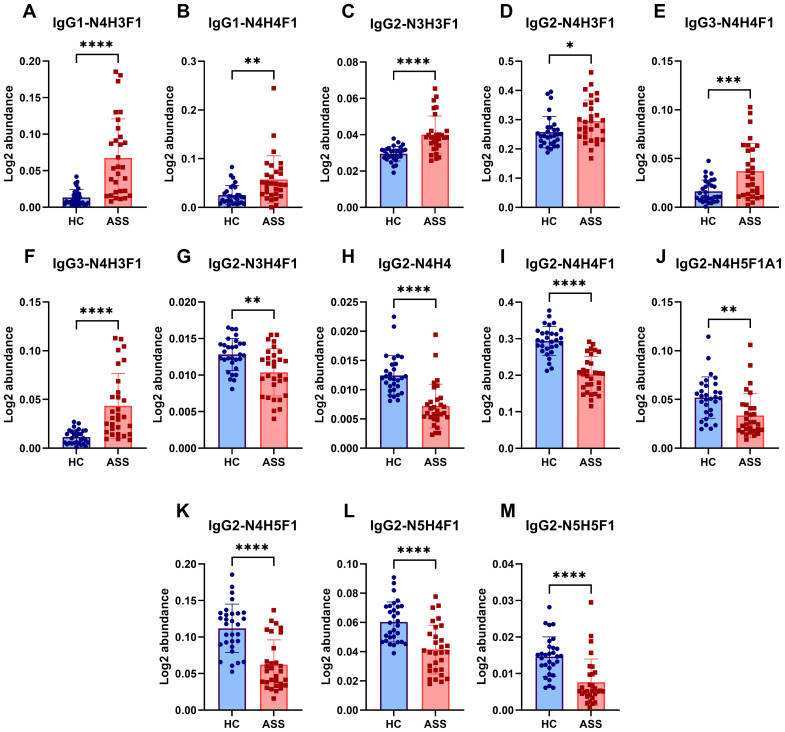
Differences in intact N-glycopeptides of IgG between the ASS and HC groups. **(A)** IgG1-N4H3F1, **(B)** IgG1-N4H4F1, **(C)** IgG2-N3H3F1, **(D)** IgG2-N4H3F1, **(E)** IgG3-N4H4F1, **(F)** IgG3-N4H3F1, **(G)** IgG2-N3H4F1, **(H)** IgG2-N4H4, **(I)** IgG2-N4H4F1, **(J)** IgG2-N4H5F1A1, **(K)** IgG2-N4H5F1, **(L)** IgG2-N5H4F1, **(M)** IgG2-N5H5F1. *p <0.05, **p<0.005, ***p<0.001, **** *p*<0.0001.

Furthermore, a total of 15 IgG intact N-glycopeptides were quantified from the two groups (**Supplementary Table S3**). The principal component analysis (PCA) plot demonstrated that these IgG intact N-glycopeptides could effectively distinguish between the ASS and HC groups (**Supplementary Figure S2**). Heatmap analysis revealed differences in intact N-glycopeptides between the ASS and HC groups (**Supplementary Figure S3**). Upon further analysis of the intact N-glycopeptides of the IgG subclasses, 13 differentially expressed intact N-glycopeptides were detected (**Figure 4**). The results indicated that six intact N-glycopeptides were significantly increased (**Figures 4A-F**). Another set of 7 intact N-glycopeptides was significantly decreased (**Figures 4G-M**). Additionally, the levels of IgG2-N4H4F1A1 and IgG2-N5H3F1 did not significantly differ between the two groups (**Supplementary Figure S4**). The diverse IgG subclasses, namely, IgG1, IgG2, IgG3, and IgG4, display significant variations in their biological activities. This includes the capacity for complement fixation (IgG3 >IgG1 >IgG2 >IgG4) as well as the ability to bind to Fc receptors. Among these subclasses (34), IgG2 is distinguished by its somewhat restricted capacity in terms of complement and FC receptor binding. Our study revealed that seven intact N-glycopeptides were derived from the IgG2 subclass. Nevertheless, individuals who are deficient in certain immunoglobulin subclasses appear to be capable of making up for it with the remaining set of antibodies used to maintain overall health (35). Therefore, we hypothesize that the reduction in glycosylation of IgG2 in ASS patients might be associated with its transformation into other subclasses. However, further research is needed to confirm this hypothesis.

### IgG glycosylation patterns associated with the clinical manifestations of ASS

**Figure 5:**
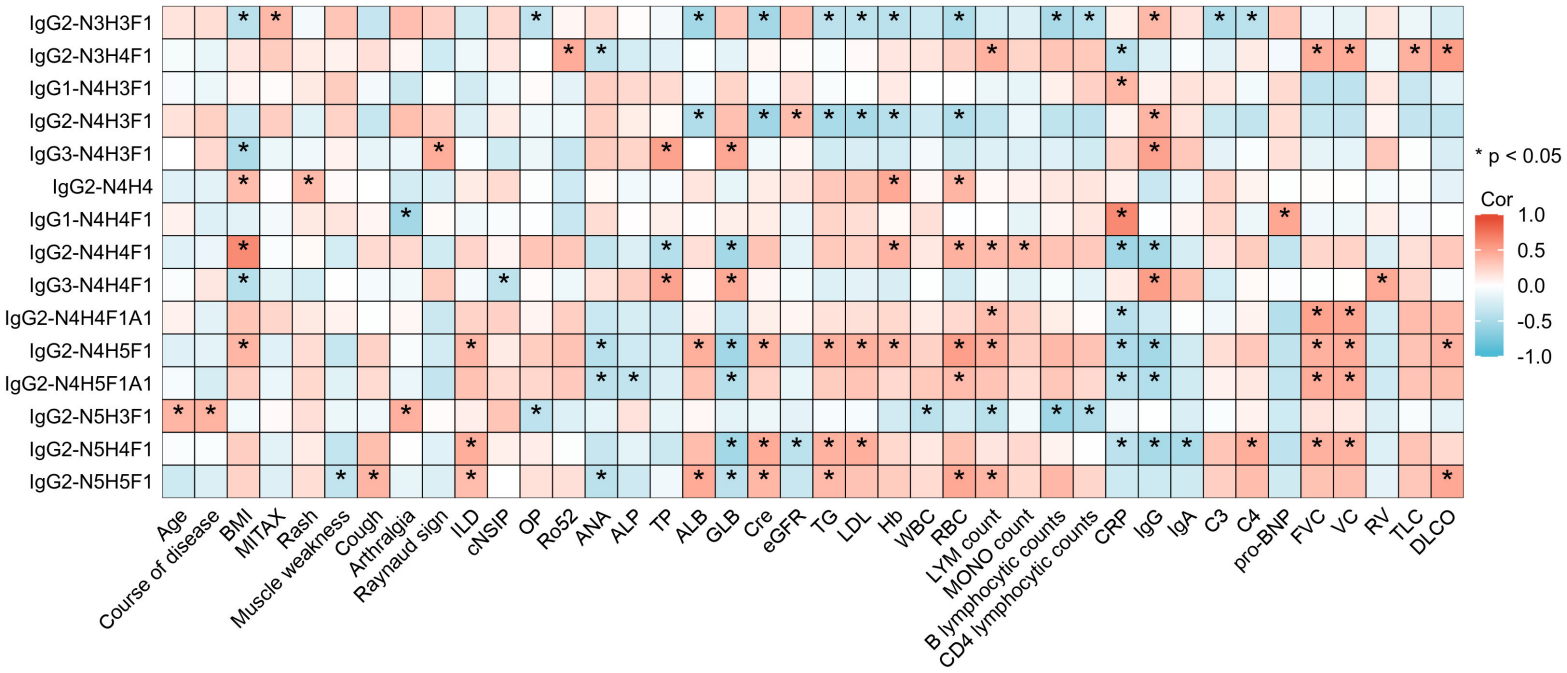
Heatmap depicting the correlation between IgG intact N-glycopeptides and the clinical manifestations of ASS. *Indicates a statistically significant correlation between the N-glycopeptide and the clinical indicators in ASS patients, with *p <0.05. BMI, bode-mass index; MITAX, myositis intention to treat activity index; ILD, interstitial lung disease; cNSIP, cell nonspecific interstitial pneumonia; OP, organizing pneumonia; Ro52, anti-cytoplasmic ribonucleoprotein of 52 kDa; ANA, antinuclear antibody; ALP, alkaline phosphatase; TP, total protein; ALB, albumin; GLB, globulin; Cre, creatinine; eGFR, estimated glomerular filtration rate; TG, triglyceride; LDL, low-density lipoprotein; Hb, hemoglobin; WBC, white blood cell count; RBC, red blood cell count; LYM, lymphocyte; MONO, monocyte; CRP, C-reactive protein; IgG, immunoglobulin G; IgA, immunoglobulin A; C3, complement C3; C4, complement C4; pro-BNP, pro-brain natriuretic peptide; FVC, forced vital capacity; VC, vital capacity; RV, residual volume; TLC, total lung capacity; DLCO, diffusing capacity of the lung for carbon monoxide.

To explore the potential clinical applications of various glycosylation patterns, we conducted a correlation analysis between the clinical manifestations and IgG intact N-glycopeptides in ASS patients. As shown in **Figure 5**, **Supplementary Tables S4**, and **S5**, our finding that N-glycosylation is significantly associated with specific clinical manifestations (such as rash, muscle weakness, Raynaud’s phenomenon, and arthralgia), disease activity, and even the development of interstitial lung disease in ASS patients implies a potential role for glycosylation in the pathogenesis and progression of this disease. The multivariate adjustment of clinical parameters that showed strong associations with two or more glycosylation features revealed persistent correlations. Nineteen clinical outcomes maintained their significance (**Supplementary Table S6**), although the effect sizes were reduced compared with the univariate findings, and no binary outcome associations were found to be significant. Previous research has revealed a correlation between the JO-1 titer and the severity and prognosis of ASS disease (36, 37). Moreover, studies have reported elevated fucosylation levels in JO-1 positive patients compared with both healthy controls and JO-1 negative patients (25), a finding that aligns with our own study. These findings suggest that the influence of the JO-1 titer on ASS disease activity might be related to glycosylation. Currently, the relationship between clinical manifestations and glycosylation in autoimmune diseases has been most extensively studied in rheumatoid arthritis. Studies have demonstrated that glycosylation plays a role in synovitis in rheumatoid arthritis and is correlated with disease activity (38). Consequently, exploring glycosylation is crucial for the monitoring of disease activity and potentially for the treatment of ASS.

Our study has certain limitations. First, the relatively small sample size makes it impossible to conduct in-depth group discussions among ASS patients with different ARSs. Second, since the theoretical sequence and structure of N-glycosylation are based on currently known biosynthetic rules and reverse synthesis strategies, it is possible that the glycopeptide structures we identified are not comprehensive enough (39). Third, our research lacks validation, even though numerous studies have been carried out, the functions of these glycopeptides are still somewhat of a mystery, and their potential clinical application value demands further validation through additional fundamental research. In the future, relevant ASS animal models or *in vitro* models, such as cell lines expressing ASS-associated autoantigens, can be used to further explore the functional significance of these glycosylation changes. Additionally, the glycosylation map of anti-ARS is a highly meaningful research direction, and research on this topic might explain the relationship of anti-ARS with the pathogenesis of ASS.

## Conclusion

In this study, we revealed distinct IgG N-glyco-signatures that differentiate ASS patients from HCs. Notable disease-specific changes included elevated fucosylation (*p*<0.0001) and reduced N-acetylneuraminic acid (*p*<0.05). Furthermore, distinct intact N-glycopeptides of IgGs were found to be correlated with pulmonary function metrics and inflammatory markers. These preliminary findings highlight specific IgG glycosylation alterations in ASS, suggesting potential links between certain glycosylation characteristics and clinical parameters associated with the disease.

## Data Availability

The datasets presented in this study can be found in online repositories. The names of the repository/repositories and accession number(s) can be found below: http://www.proteomexchange.org/, PXD058077.
