# Supplementary material for: N-glycosylation patterns of plasma immunoglobulin G in anti-synthetase syndrome disease
Source: Front Immunol. 2025 Jun 18;16:1538219. doi: 10.3389/fimmu.2025.1538219 (PMC12214897; doi:10.3389/fimmu.2025.1538219)
Supplement: Supplementary file 1 [file DataSheet1.doc]

**Supplementary Information**

**N-glycosylation patterns of plasma** **immunoglobulin G in anti-synthetase syndrome disease**

Jing Zhao^#^, Yanhong Li^#^, Yingying Ling^#^, Tong Wu, Yinlan Wu, Chunyu Tan, Lu Cheng, Deying Huang, Yi Liu*, Yong Zhang*

Department of Rheumatology and Immunology, Laboratory of Rheumatology and Immunology, Institutes for Systems Genetics, West China Hospital, Sichuan University, Chengdu 610041, China.

^#^These authors contributed equally.

*Corresponding authors:

Yong Zhang (nankai1989@foxmail.com/zhangyong0809@wchscu.cn), Yi Liu (yiliu8999@wchscu.cn)

**Table of Contents**

**Supplementary Figure S1.** Undifferentiated intact IgG N-glycans in the HC and ASS groups.

**Supplementary Figure S2.** PCA plot shows that these IgG intact N-glycopeptides can effectively distinguish between the HC and ASS groups.

**Supplementary Figure S3.** Heatmap analysis of IgG intact N-glycopeptides from the ASS and HC groups.

**Supplementary Figure S4.** Undifferentiated intact N-glycopeptides in the HC and ASS groups.

**Supplementary Table S1.** Baseline demographics and clinical characteristics of all individuals.

**Supplementary Table S2.** Quantitative result of IgG N-glycans in the two groups.

**Supplementary Table S3.** Quantitative result of IgG intact N-glycopeptides in the two groups.

**Supplementary Table S4.** Correlation between IgG glycosylation and clinical features of ASS.

**Supplementary Table S5.** Correlation p-value between intact N-glycopeptides and clinical features of ASS.

**Supplementary Table S6.** Multiple linear regression results between intact N-glycopeptides and clinical features of ASS.

**Supplementary Figure S1.** Undifferentiated intact IgG N-glycans in the HC and ASS groups.


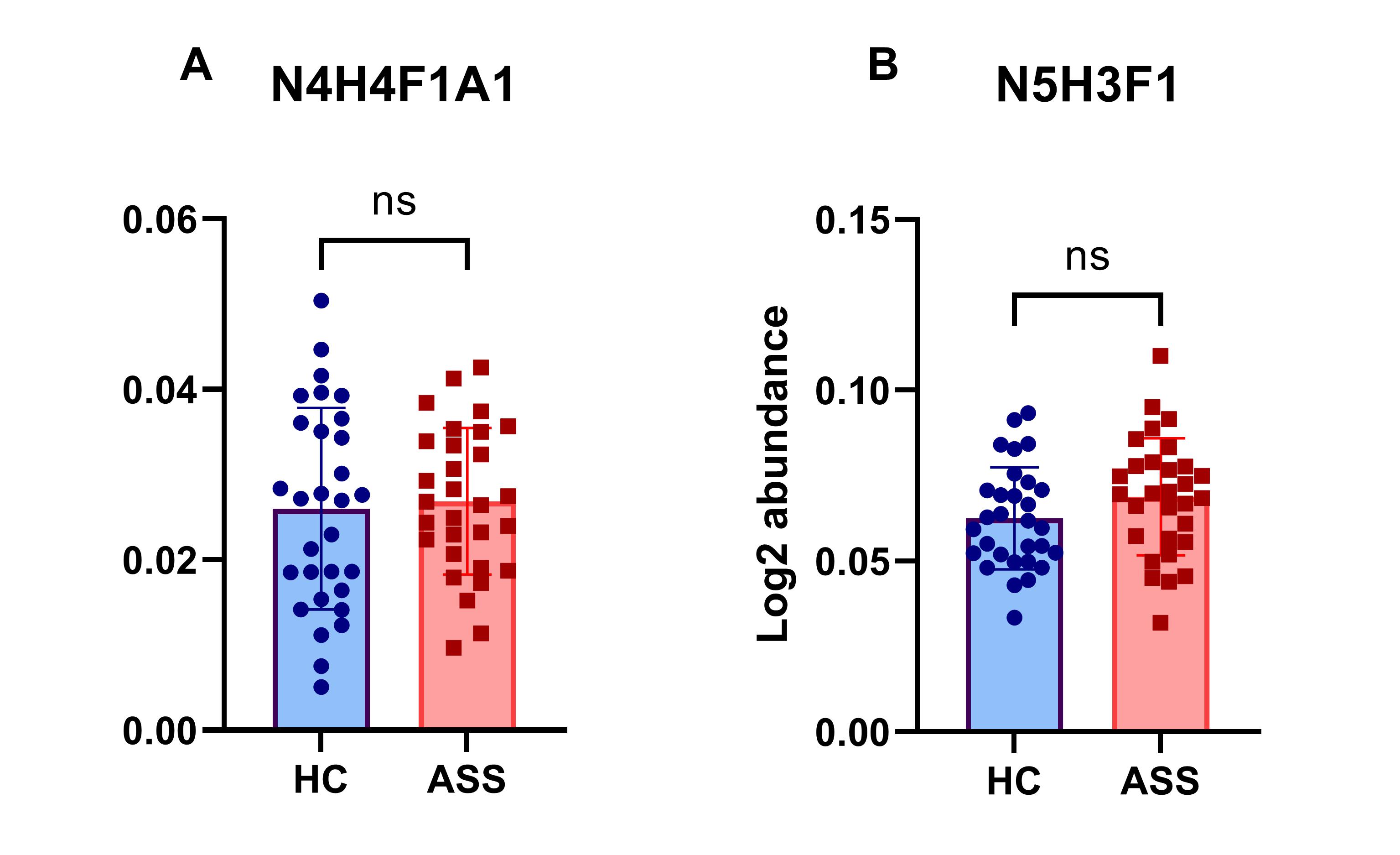


**Supplementary Figure S2.** PCA plot shows that these IgG intact N-glycopeptides can effectively distinguish between the HC and ASS groups.


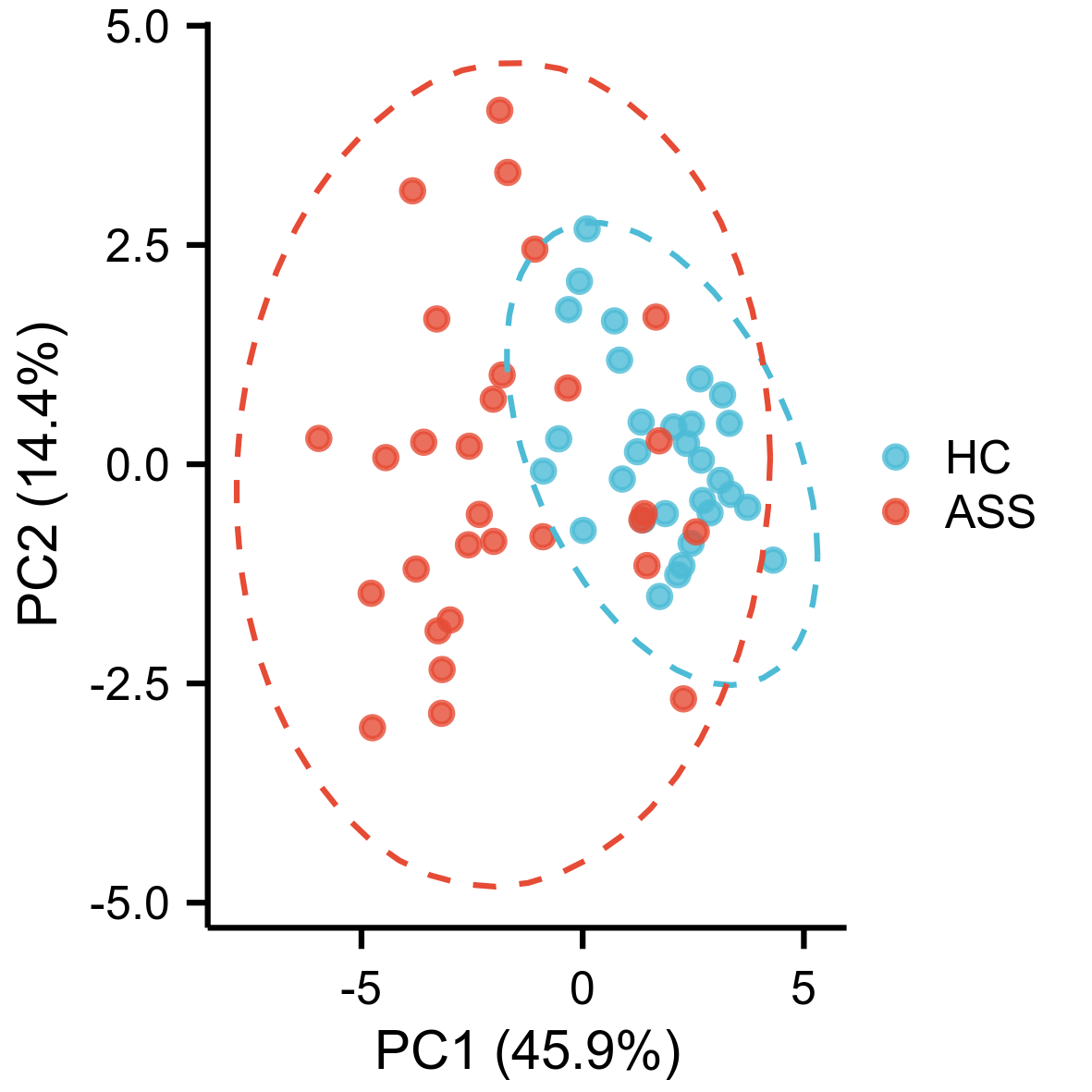


**Supplementary Figure S3.** Heatmap analysis of IgG intact N-glycopeptides from the ASS and HC groups.

**
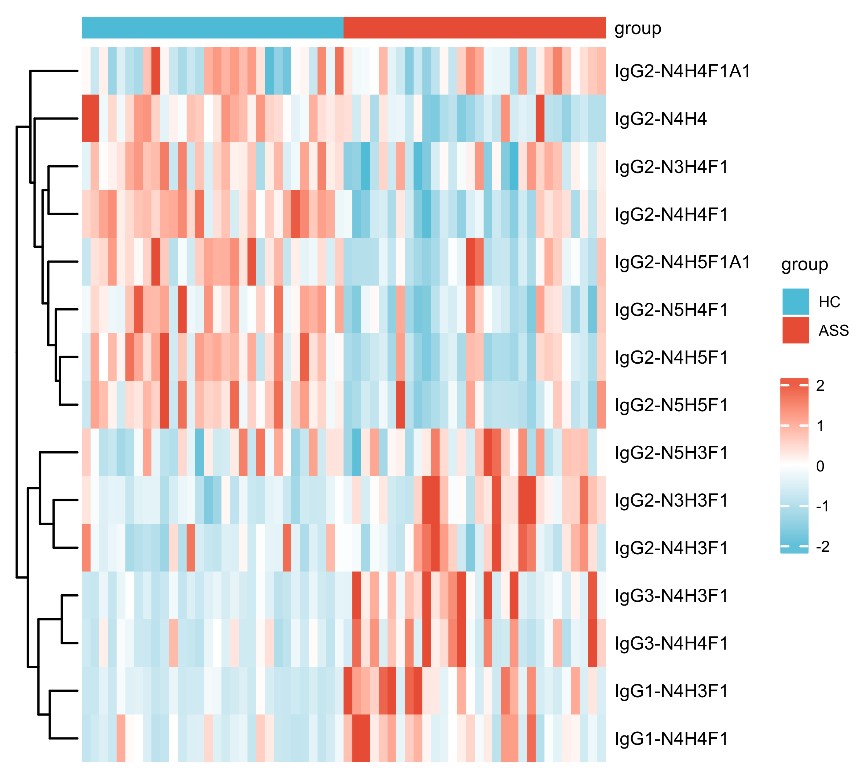
**

**Supplementary Figure S4.** Undifferentiated intact N-glycopeptides in the HC and ASS groups.

**
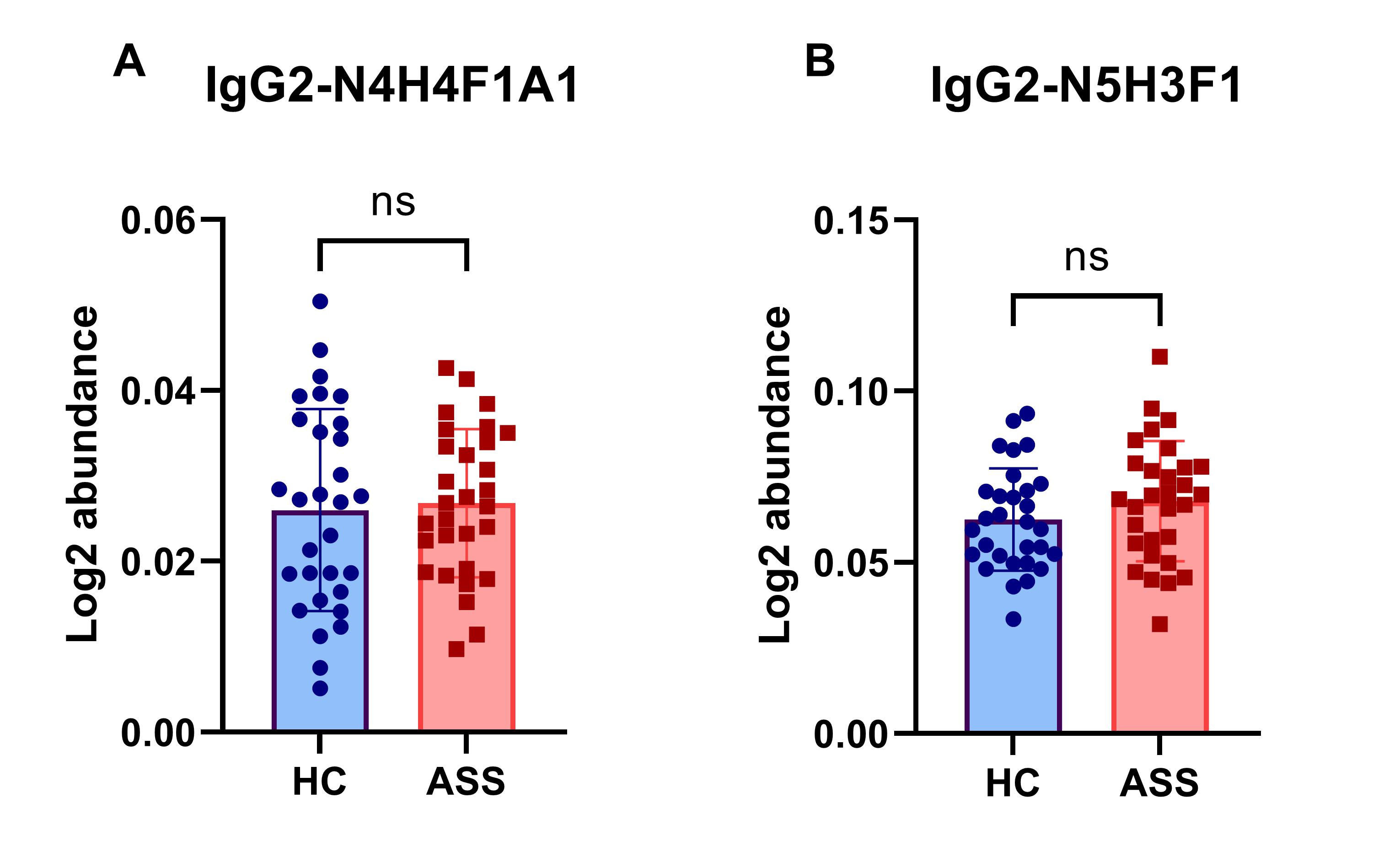
**
